# ﻿Two new species of *Nigropunctata* and the first report of sexual morph of *Melanographium citri* (Pallidoperidiaceae, Xylariales) from south-western China

**DOI:** 10.3897/mycokeys.122.161215

**Published:** 2025-09-17

**Authors:** Chang-Tao Lu, Yu-Lin Ren, Kamran Habib, Qin-Fang Zhang, Li-Li Liu, Ji-Chuan Kang, Xiang-Chun Shen, Nalin N. Wijayawardene, Salim S. Al-Rejaie, Chanhom Loinheuang, Qi-Rui Li, Qing-De Long, Abdallah M. Elgorban

**Affiliations:** 1 State Key Laboratory of Discovery and Utilization of Functional Components in Traditional Chinese Medicine & School of Pharmaceutical Sciences, Guizhou Medical University, Gui’an New District, Guizhou, 561113, China; 2 The High Efficacy Application of Natural Medicinal Resources Engineering Centre of Guizhou Province (The Key Laboratory of Optimal Utilization of Natural Medicine Resources), Guizhou Medical University, Gui’an New District, Guizhou, 561113, China; 3 Department of Botany, Khushal Khan Khattak University, Karak, KP, 27200 Pakistan; 4 Department of Entomology and Plant Pathology, Faculty of Agriculture, Chiang Mai University, Chiang Mai 50200, Thailand; 5 Engineering and Research Centre for Southwest Bio-Pharmaceutical, Resources of National Education Ministry of China, Guizhou University, Guiyang, Guizhou, 550025, China; 6 Center for Yunnan Plateau Biological Resources Protection and Utilization, College of Biology and Food Engineering, Qujing Normal University, Qujing, Yunnan 655011, China; 7 Departmental of Pharmacology and Toxicology, College of Pharmacy, King Saud University, P.O. Box 55760, 11451 Riyadh, Saudi Arabia; 8 Department of Biology, Faculty of Natural Sciences, National University of Laos, Vientiane Capital, Lao People's Democratic Republic; 9 Center of Excellence in Biotechnology Research (CEBR), DSR, King Saud University, P.O. Box. 2454 Riyadh, Saudi Arabia

**Keywords:** Ascomycetes, bambusicolous fungi, karst ecosystem, systematics

## Abstract

In our ongoing survey of xylarialean fungi in southern China, we obtained specimens of *Nigropunctata* and *Melanographium* from bamboo and palm, respectively. Maximum Likelihood and Bayesian Inference analyses, based on five loci (ITS, LSU, *rpb2*, *tub2* and *tef-1α*), were used to clarify the taxonomic placement of these specimens. Our results confirm that the specimens represent two new species *Nigropunctata shiwandashanensis* and *N. puerzhenensis*, as well as a new geographical record, *Melanographium citri*, which represents the first report of the sexual state for this genus. We provide detailed morphological descriptions and illustrations, as well as a comparison between the new species and related or similar species.

## ﻿Introduction

The genus *Nigropunctata* M.C. Samar. & K.D. Hyde was established by Samarakoon et al. (2022) to accommodate three species *N. bambusicola* (designated as the type species), *N. nigrocircularis* and *N. thailandica*. Initially, the genus was classified as *incertae sedis* within the order Xylariales. Later, multi-locus phylogenetic analyses (ITS, LSU, *tub2*, *rpb2*, *tef-1α*) by Sugita et al. (2024) demonstrated that *Nigropunctata*, along with the genera *Amphigermslita*, *Crassipseudostroma*, *Minuticlypeus* and *Pallidoperidium*, forms a distinct monophyletic clade within Xylariales. As a result, the family Pallidoperidiaceae was introduced to accommodate this lineage.

Species of *Nigropunctata* are characterised by immersed, solitary or scattered ascomata appearing as small black dots, unitunicate cylindrical asci with a J+ discoid or inverted hat-shaped apical ring and cylindrical to broadly ellipsoidal ascospores, surrounded by a thick mucilaginous sheath or lack, with a straight germ slit running the entire spore length (Samarakoon et al. 2022). Morphological variation amongst *Nigropunctata* species is minimal, which complicates species delimitation, based solely on phenotype. Therefore, species delineation within the genus necessitates an integrative approach combining detailed morphological characterisation with multilocus DNA sequence data. Currently, the genus comprises eleven species *N. bambusicola*, *N. chiangraiensis*, *N. chinensis*, *N. complanata*, *N. conspicua*, *N. hydei*, *N. khalidii*, *N. liuzhouensis*, *N. nigrocircularis*, *N. saccata* and *N. thailandica*. All species are associated with bamboo hosts and have been primarily reported from China and Thailand (Samarakoon et al. 2022; Samarakoon et al. 2023; Li et al. 2024; Sugita et al. 2024; Tun et al. 2024; Cao et al. 2025; Habib et al. 2025).

The genus *Melanographium* Sacc. was established by Saccardo (1913) with *M. spleniosporum* as the type species. Ellis (1963) transferred *Trichosporum selenioides* Sacc. & Paol. to *Melanographium* as *M. selenioides* (Sacc. & Paol.) M.B. Ellis and synonymised *M. spleniosporum* under this epithet. Consequently, *M. selenioides* was recognised as the type species of the genus (Ellis 1963). Species of *Melanographium* are characterised by dark, unbranched conidiophores emerging from immersed stromata, polyblastic conidiogenous cells with sympodial proliferation and pigmented, 1-celled, often reniform conidia. Species within the genus are differentiated primarily based on conidiophore structure and grouping, as well as conidial morphology. Currently, 15 species are accepted within the genus including *M. anceps*, *M. calami*, *M. citri*, *M. cookei*, *M. indicum*, *M. laxum*, *M. maximum*, *M. palmicola*, *M. phoenicis*, *M. proliferum*, *M. selenioides*, *M. smilacis*, *M. spleniosporum*, *M. thunbergiae* and *M. trachycarpi*. Most of these species have been isolated from palm hosts (Ellis 1963; Srivastava and Morgan-Jones 1996; Goh and Hyde 1997; Somrithipol and Jones 2005; Hyde et al. 2020; Boonmee et al. 2021). Hyde et al. (2020) provided the first molecular data for the genus, based on a new species, *M. phoenicis*. Boonmee et al. (2021) introduced *M. smilacis* with supporting molecular data. Zhang et al. (2024) recognised *M. selenioides*, *M. citri* and *M. palmicola* and designated relevant reference specimens. To date, no sexual morph has been reported for any species within *Melanographium*.

In this study, we introduce two new *Nigropunctata* species, *N. puerzhenensis* and *N. shiwandashanensis* and report the first documented sexual state for the genus *Melanographium*, found in *M. citri*. Multi-locus phylogeny (ITS, LSU, *tub2*, *rpb2* and *tef-1α*) supports their taxonomic placements, while morphological comparisons distinguish them from closely-related species.

## ﻿Materials and methods

### ﻿Sample collection

The specimens were collected during surveys conducted in Yunnan Province and Guangxi Zhuang Autonomous Region in China during the period July-November 2024. All related habitat information was recorded. Materials were placed in paper bags and taken to the lab for morphological characterisation and isolation. To preserve the freshness of the specimens, they were dried using a portable fan dryer. The dried specimens were carefully labelled and stored. After this preparation, the specimens were ready for both morphological and molecular studies. All specimens were deposited at the Herbarium of Guizhou Medical University (**GMB**) and the Herbarium of Cryptogams, Kunming Institute of Botany, Chinese Academy of Sciences (**KUN-HKAS**), living cultures being deposited at the Guizhou Medical University Culture Collection (**GMBC**). All taxonomic names used in the text are described in strict accordance with Index Fungorum and MycoBank; hence, no authorities and years of publications are given in the text apart from the taxonomic entries.

### ﻿Morphological characterisation and isolation

Macroscopic features (ostiole, clypeus etc.) of the specimens were examined using an Olympus SZ61 stereomicroscope and photographed using a Canon 700D digital camera. Microscopic morphological features (ascomata, peridium, paraphyses, asci, ascospores etc.) were observed using an optical microscope (Nikon Ni) and photographed using an attached Canon 700D digital camera. Melzer’s iodine reagent was used to test the apical apparatus structures for amyloid reaction. Asci and ascospores of the samples were measured using Tarosoft Image Framework (v.0.9.0.7). Images were polished using Adobe Photoshop CS6 (Adobe Systems, USA). Pure cultures were obtained by single-ascospore isolation (Liu et al. 2025) and maintained at 25 °C for 1–5 weeks on PDA (potato dextrose agar) and oatmeal-agar (OA) medium.

### ﻿DNA extraction, PCR amplification and sequencing

Mycelium was scraped from pure culture plates using a sterilised scalpel and was used for DNA extraction with the methods following the manufacturer’s instructions of the BIOMIGA fungus genomic DNA extraction kit. For some specimens where the ascospores did not germinate, we used a method of directly extracting DNA from the contents of the perithecium. The DNA samples were kept at –20 °C. Internal transcribed spacers (ITS), large subunit LSU, β-tubulin (*tub2*), RNA polymerase II second largest subunit (*rpb2*) and translation elongation factor (*tef-1α*) were amplified by PCR with primers ITS1/ITS4 (White et al. 1990; Gardes and Bruns 1993), LR0R/LR5 (Vilgalys and Hester 1990), Bt2a/Bt2b (Glass and Donaldson 1995), rpb2-5f/7cR (Liu et al. 1999) and EF1 983F/EF1-2218R (Rehner and Buckley 2005), respectively. The components of a 25 μl volume PCR mixture was: 9.5 μl of double distilled water, 12.5 μl of PCR Master Mix, 1 μl of each primer and 1 μl of template DNA. Qualified PCR products were checked through 1.5% agarose gel electrophoresis stained with GoldenView and were sent to Sangon Co., China, for sequencing.

### ﻿Sequence alignments and phylogenetic analyses

All the obtained sequences were deposited in the GenBank (Table 1). These sequences were compared with each other and all the known sequences in GenBank using the BLASTn algorithm for precise identification. The molecular phylogeny was inferred from a combined dataset of ITS, LSU, *tub2*, *rpb2* and *tef-1α* sequences. The reference sequences retrieved from open databases originated from recent published literature and the BLASTn results from close matches. Sequences were aligned using the MAFFT v.7.110 online programme (Katoh et al. 2019) with the default settings, respectively. The alignment was adjusted manually using BioEdit v.7.0.5.3 (Hall 1999) where necessary. The Maximum Likelihood (ML) analysis was implemented in RAxML v.8.2.12 using the GTRGAMMA substitution model with 1,000 bootstrap replicates (Stamatakis 2014). The phylogenetic analyses were also performed for Bayesian Inference in MrBayes v. 3.2.2 (Ronquist et al. 2012) online. The Markov Chain Monte Carlo (MCMC) sampling in MrBayes v.3.2.2 (Ronquist et al. 2012) was used to determine the posterior probabilities (PP). Six simultaneous Markov chains were run for 1,000,000 generations and trees were sampled every 1,000^th^ generation. The phylogenetic tree was visualised in FigTree v.1.4.4 (Rambaut 2018). All analyses were run on the CIPRES Science Gateway v. 3.3 webportal (Miller et al. 2010).

**Table 1.** Taxa and corresponding GenBank accession numbers of sequences used in the phylogenetic analysis of Fig. 1. Notes: Type specimens are marked with T; “N/A”: indicates no sequence available in GenBank; newly-generated sequences are indicated in bold.

Species	Voucher	GenBank Accession Numbers					References
		ITS	LSU	rpb2	tub2	tef-1a	
*Amphigermslita deformis*	HHUF 30660^T^	LC760553	LC760572	LC760592	N/A	LC760605	Sugita et al. (2024)
*Amphigermslita deformis*	HHUF 30661	LC760554	LC760573	LC760593	N/A	LC760606	Sugita et al. (2024)
*Amphigermslita fusiformis*	HHUF 30663^T^	LC760555	LC760574	LC760594	N/A	LC760607	Sugita et al. (2024)
*Amphigermslita fusiformis*	HHUF 30664	N/A	LC760575	LC760595	N/A	LC760608	Sugita et al. (2024)
*Amphigermslita pseudofusiformis*	HHUF 30662^T^	LC760556	LC760576	LC760596	N/A	LC760609	Sugita et al. (2024)
*Amphigermslita subyunnanensis*	GMB1153^T^	PP133235	PQ860488	N/A	PP209124	N/A	Li et al. (2024)
*Amphigermslita yunnanensis*	HKAS 122747^T^	OQ158966	OQ170888	N/A	N/A	N/A	Ren et al. (2024)
*Anthostomella lamiacearum*	MFLU 18-0101^T^	MW240669	MW240599	MW658648	N/A	N/A	Samarakoon et al. (2022)
*Anthostomella guangxiensis*	GMB5609^T^	PQ884707	PQ885419	N/A	PQ893603	N/A	Liu et al. (2025)
*Anthostomella guangxiensis*	GMB5616	PQ884708	PQ885420	N/A	PQ893604	N/A	Liu et al. (2025)
*Anthostomella leucobasis*	GMB1143	PP153382	N/A	PP198092	PP203030	N/A	Li et al. (2024)
*Crassipseudostroma phyllostachydis*	HHUF 30678^T^	LC760557	LC760577	LC760597	N/A	LC760610	Sugita et al. (2024)
*Gyrothrix encephalarti*	CBS 146684^T^	NR_170834	MT373358	ON399342	N/A	MT375117	Crous et al. (2020)
*Gyrothrix encephalarti*	CBS 114517	ON400755	ON400808	ON399314	N/A	N/A	Hernández-Restrepo et al. (2022)
*Gyrothrix encephalarti*	CBS 114515	N/A	ON400809	ON399315	N/A	N/A	Hernández-Restrepo et al. (2022)
*Gyrothrix verticillata*	CBS 148806	ON400759	ON400813	ON399318	N/A	N/A	Hernández-Restrepo et al. (2022)
*Gyrothrix verticillata*	CBS 148805	ON400760	ON400814	ON399319	N/A	N/A	Hernández-Restrepo et al. (2022)
***Melanographium citri***	**GMB4902**	**PV933306**	**PV933312**	**PV933947**	**PV933949**	**PV933941**	**The study**
***Melanographium citri***	**GMB4951**	**PV933309**	**PV933315**	**PV933948**	**PV933954**	**PV933942**	**The study**
*Melanographium citri*	SNC92	PP592412	PP621039	PP780226	N/A	PP740449	Zhang et al. (2024)
*Melanographium palmicola*	SNC154	PP592414	PP621041	PP780228	PP816196	PP740451	Zhang et al. (2024)
*Melanographium phoenicis*	MFLUCC 18-1481^T^	MN482677	MN482678	N/A	N/A	MN481518	Hyde et al. (2020)
*Melanographium selenioides*	SNC142	PP592415	PP621042	N/A	N/A	PP740452	Zhang et al. (2024)
*Melanographium smilacis*	MFLU 21-0075^T^	NR_177581	NG_088273	N/A	N/A	MZ567091	Boonmee et al. (2021)
*Melanographium* sp.	HKAS 131232	OR813920	OR813921	N/A	N/A	N/A	Unpublished
*Minuticlypeus biconcavus*	GMB6221^T^	PQ874038	PQ860484	N/A	N/A	PQ826978	Habib et al. (2025)
*Minuticlypeus biconcavus*	GMB6222	PQ874039	PQ860485	N/A	N/A	PQ826979	Habib et al. (2025)
*Minuticlypeus discosporus*	HHUF 30673^T^	LC760559	LC760579	N/A	N/A	LC760612	Sugita et al. (2024)
*Minuticlypeus discosporus*	HHUF 30672	LC760558	LC760578	LC760598	N/A	LC760611	Sugita et al. (2024)
*Minuticlypeus rhaphidophylli*	GMB1150	PP153386	PQ860487	N/A	PP203034	N/A	Li et al. (2024)
*Minuticlypeus xiaohensis*	GMB4503^T^	PQ066510	PQ066518	N/A	PQ083530	PQ083532	Zhou et al. (2024)
*Minuticlypeus xiaohensis*	GMB4552	PQ066511	PQ066519	N/A	PQ083531	PQ083533	Zhou et al. (2024)
*Minuticlypeus yunnanensis*	GMB5631^T^	PQ884705	PQ885417	N/A	N/A	N/A	Liu et al. (2025)
*Minuticlypeus yunnanensis*	GMB5638	PQ884706	PQ885418	N/A	PQ893602	N/A	Liu et al. (2025)
*Nigropunctata bambusicola*	MFLU 19-2134	MW240662	MW240592	MW658644	N/A	MW759547	Samarakoon et al. (2022)
*Nigropunctata bambusicola*	MFLU 19-2145^T^	MW240664	MW240594	MW658646	N/A	MW759548	Samarakoon et al. (2022)
*Nigropunctata chiangraiensis*	MFLUCC 23-0238^T^	OR909712	N/A	OR757300	PQ397547	PQ505632	Tun et al. (2024)
*Nigropunctata chinensis*	GMB6223^T^	PQ874034	PQ860480	PQ826932	PQ863998	PQ826974	Habib et al. (2025)
*Nigropunctata chinensis*	GMB6224	PQ874035	PQ860481	PQ826933	PQ863999	PQ826975	Habib et al. (2025)
*Nigropunctata complanata*	HHUF 30675^T^	LC760561	LC760581	LC760600	N/A	LC760614	Sugita et al. (2024)
*Nigropunctata complanata*	HHUF 30677	LC760563	LC760583	LC760602	N/A	LC760616	Sugita et al. (2024)
*Nigropunctata conspicosa*	MFLU 24-0128^T^	PP868305	PP868306	PP869337	N/A	PP869336	Cao et al. (2025)
*Nigropunctata hydei*	CMUB40018^T^	OR507150	OR507163	OR504422	N/A	N/A	Samarakoon et al. (2023)
*Nigropunctata hydei*	MFLU 23- .00410	OR507151	OR507164	N/A	N/A	N/A	Samarakoon et al. (2023)
*Nigropunctata khalidii*	GMB1156^T^	PP153389	N/A	N/A	PP209114	N/A	Li et al. (2024)
*Nigropunctata liuzhouensis*	GMB6225^T^	PQ874036	PQ860482	N/A	PQ864000	PQ826976	Habib et al. (2025)
*Nigropunctata liuzhouensis*	GMB6226	PQ874037	PQ860483	N/A	PQ864001	PQ826977	Habib et al. (2025)
*Nigropunctata nigrocircularis*	MFLU 19-2130^T^	MW240661	MW240591	N/A	MW775612	MW759546	Samarakoon et al. (2022)
***Nigropunctata puerzhenensis***	**GMB4904^T^**	**PV933307**	**PV933313**	**N/A**	**PV933952**	**PV933943**	**The study**
***Nigropunctata puerzhenensis***	**GMB4953**	**PV933310**	**PV933316**	**N/A**	**PV933955**	**PV933945**	**The study**
*Nigropunctata saccata*	MFLU 19-2144^T^	MW240663	MW240593	MW658645	MW775613	N/A	Samarakoon et al. (2023)
***Nigropunctata shiwandashanensis***	**GMB4903^T^**	**PV933308**	**PV933314**	**PV933949**	**PV933953**	**PV933944**	**The study**
***Nigropunctata shiwandashanensis***	**GMB4952**	**PV933311**	**PV933317**	**PV933950**	**PV933956**	**PV933946**	**The study**
*Nigropunctata thailandica*	MFLU 19-2118^T^	MW240659	MW240589	MW658643	N/A	MW759544	Samarakoon et al. (2022)
*Pallidoperidium exasperatum*	HHUF 30174^T^	LC760564	LC760584	LC760603	N/A	LC760617	Sugita et al. (2024)
*Pallidoperidium exasperatum*	HHUF 30667	LC760567	LC760587	N/A	N/A	LC760620	Sugita et al. (2024)
*Pallidoperidium paraexasperatum*	HHUF 30668^T^	LC760568	LC760588	N/A	N/A	LC760621	Sugita et al. (2024)
*Pallidoperidium paraexasperatum*	HHUF 30671	LC760571	LC760591	LC760604	N/A	LC760624	Sugita et al. (2024)
*Pseudoceratocladium polysetosum*	CBS 129023^T^	NR_154849	NG_059024	ON399348	N/A	N/A	Hernández-Restrepo et al. (2017)
*Pseudoceratocladium polysetosum*	CBS 126092	MH864077	MH875534	ON399347	N/A	N/A	Vu et al. (2019)
*Pseudocircinotrichum papakurae*	CBS 101373	KR611876	KR611897	N/A	N/A	N/A	Crous et al. (2015)
*Pseudocircinotrichum papakurae*	CBS 140221	ON400768	ON400820	ON399349	N/A	N/A	Hernández-Restrepo et al. (2022)
*Selenodriella brasiliana*	CBS 140236	ON400770	ON400822	ON399357	N/A	N/A	Hernández-Restrepo et al. (2022)
*Selenodriella brasiliana*	CBS 140227^T^	ON400769	ON400821	ON399356	N/A	N/A	Hernández-Restrepo et al. (2022)
*Selenodriella cubensis*	CBS 683.96^T^	NR_154414	NG_058151	N/A	N/A	N/A	Hernández-Restrepo et al. (2016)
*Selenodriella fertilis*	CBS 148328	ON400772	ON400824	ON399359	N/A	N/A	Hernández-Restrepo et al. (2022)
*Selenodriella fertilis*	CPC 16273	ON400771	ON400823	ON399358	N/A	N/A	Hernández-Restrepo et al. (2022)
*Xenoanthostomella chromolaenae*	MFLUCC 17-1484^T^	MN638863	MN638848	MN648729	N/A	MN648732	Hyde et al. (2020)
*Xenoanthostomella chromolaenae*	CBS 148703	ON400785	ON400842	N/A	N/A	N/A	Hernández-Restrepo et al. (2022)

Notes: Type specimens are marked with T; “N/A”: indicates no sequence available in GenBank; newly-generated sequences are indicated in bold.

## ﻿Results

### ﻿Phylogeny

**Figure 1.**
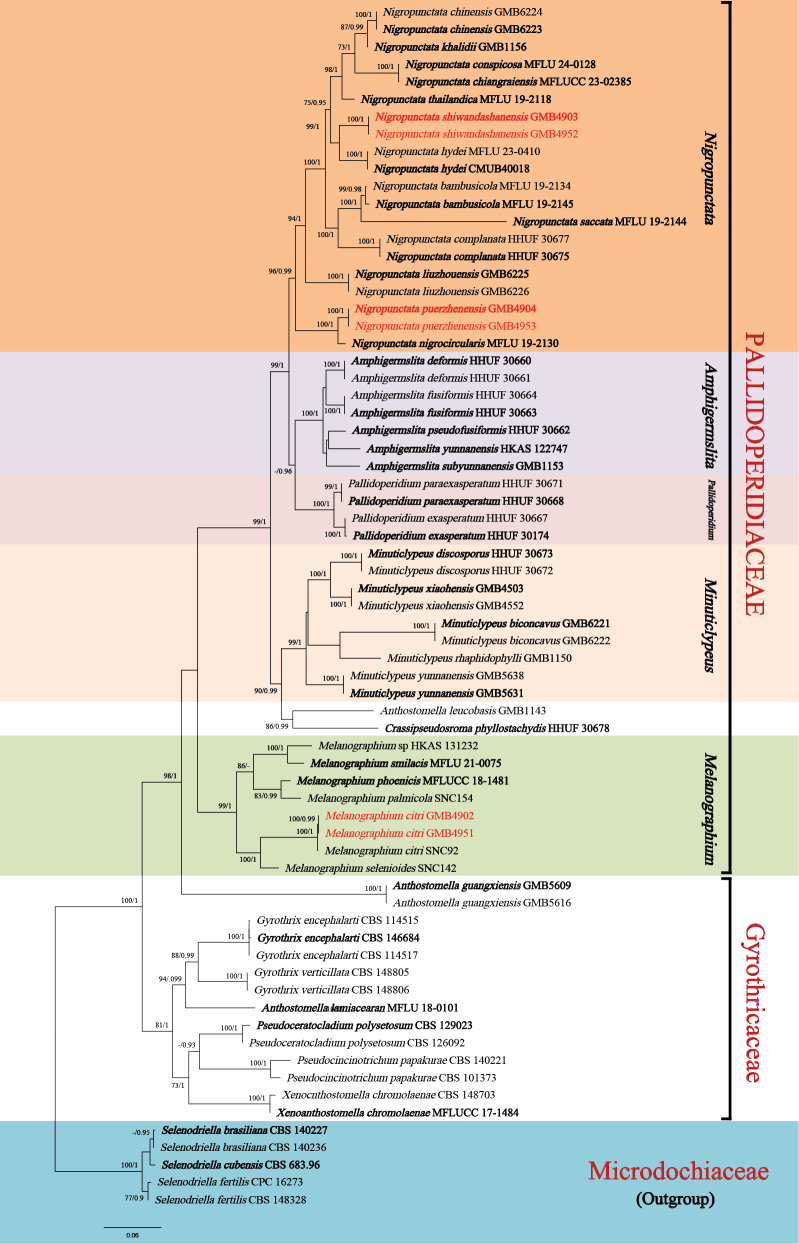
Molecular phylogenetic analysis of *Nigropunctata puerzhenensis*, *Nigropunctata shiwandashanensis*, *Melanographium citri* and related taxa, based on a combined ITS, LSU, *tub2*, *rpb2* and *tef-1α* sequence dataset. Bootstrap support values for Maximum Likelihood (ML) greater than 70% and Bayesian posterior probabilities (BPP) greater than 0.90 are displayed above or below the respective branches (ML/PP). The newly-described species are marked red. Holotype and ex-type materials are in bold.

After the exclusion of ambiguously aligned regions and long gaps, the final combined data matrix contained 4028 characters (ITS: 1–520, LSU: 521–1690, *tub2*: 1691–2105, *rpb2*: 2106–3101, *tef-1α*: 3102–4028). *Selenodriella brasiliana*, *S. cubensis* and *S. fertilis* (Microdochiaceaee) were added as the outgroup (Tun et al. 2024). Tree topology from ML analysis was similar to BI analysis. The best-scoring RAxML tree is presented in Fig. 1. In the phylogram, the sequence of our *Melanographium* collections GMB4902 and GMB4951 clustered with *M. citri* with strong support (100BS/1PP). The other two isolates grouped within the genus *Nigropunctata*. *Nigropunctata puerzhenensis* formed a well-supported sister clade (100BS/1PP) with *N. nigrocircularis*, while *N. shiwandashanensis* appeared as a sister to *N. hydei*, also with high support (91BS/1PP).

### ﻿Taxonomy

#### ﻿ Melanographium

Taxon classificationFungiXylarialesPallidoperidiaceae

Sacc., Annls mycol. 11(6): 557 (1913) emend.

34924B4B-B622-5246-B9E9-4A5BD319D90E

##### Description.

**Sexual morph**: Ascomata immersed, globose to subglobose, ostiolate. Peridium bilayered, outer dark brown, inner hyaline, cell ***textura angularis***. Paraphyses filamentous, guttulate. Asci 8-spored, unitunicate, clavate-cylindrical, short-pedicellate, apical apparatus inamyloid. Ascospores unicellular, fusiform with rounded ends, dark brown, surrounded by a distinct mucilaginous sheath, germ slit centre, ca. 1/4 of the length of the ascospore. **Asexual morph**: Conidiophore dark, unbranched, emerging from immersed stromata, macronematous, mononematous, conidiogenous cells polyblastic with sympodial proliferation. Conidia pigmented, 1-celled, often reniform.

##### Type species.

*Melanographium selenioides* (Sacc. & Paol.) M.B. Ellis = *M. spleniosporum* Sacc.

#### ﻿ Melanographium citri

Taxon classificationFungiXylarialesPallidoperidiaceae

(Gonz. Frag. & Cif.) M.B. Ellis, Mycological Papers 93: 21 (1963)

3696043B-86B6-58CB-8967-FCFC7931D550

Fig. 2

##### Description.

Saprobic on a dry palm branch. ***Sexual morph***: Ascomata 440–680 µm wide, 470–540 µm high, immersed, solitary or gregarious, appearing as small black spot, in cross-section globose to subglobose with a flattened base. Ostioles centric, slightly papillate, black, slightly higher than the surface of the host. Peridium 12–27.4 µm thick, comprised of two layers; outer layer composed of thick-walled, dark-brown, cells of ***textura angularis***; inner layer composed of thin-walled, hyaline, cells of ***textura angularis***. Paraphyses 2–5 µm (x̄ = 3 µm, n = 30) wide, longer than the asci, numerous, filamentous, guttulate. Asci 185–260 × 15–35 µm (x̄ = 216 × 23 µm, n = 30) 8-spored, unitunicate, clavate-cylindrical, short-pedicellate, apically rounded, with non-amyloid apical apparatus. Ascospores 20–27.5 × 8.5–11.5 µm (x̄ = 25 × 10 µm, n = 30), overlapping unicellular, fusiform with rounded ends, dark brown, covered with a thick mucilaginous sheath measuring 4–14.5 µm (x̄ = 8.0 µm, n = 30), with a straight germ slit on the centre, ca. 1/4 of the length of the spore. **Asexual morph**: not found.

**Figure 2.**
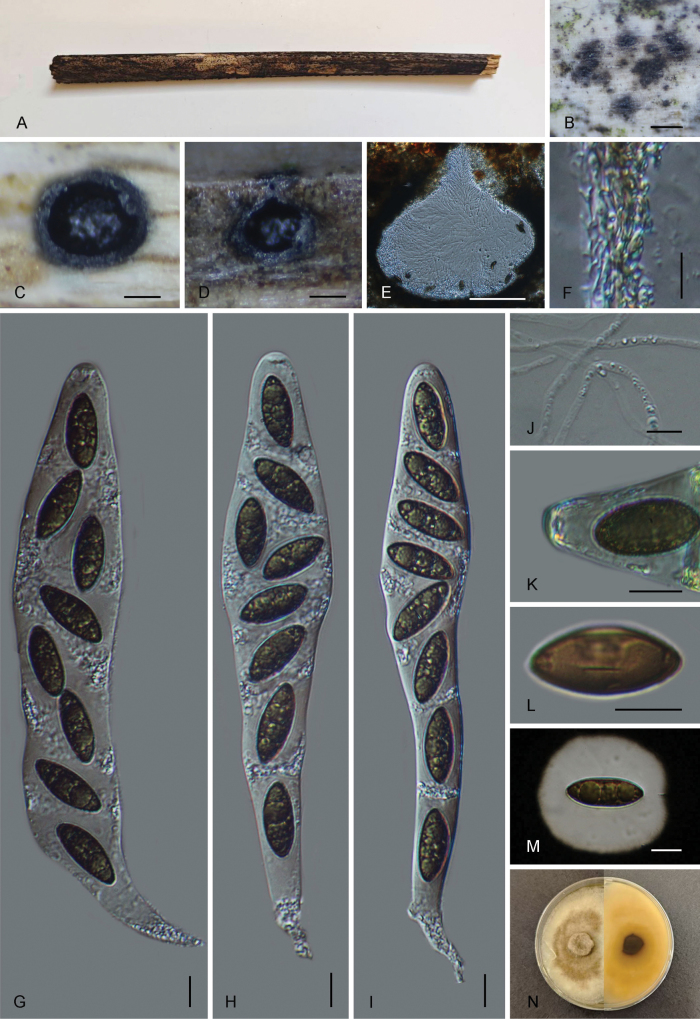
*Melanographium citri* (GMB4902). A. Material; B. Ascomata on the surface of host; C. Cross-section of ascoma; D, E. Longitudinal sections of ascoma; F. Peridium; G–I. Asci with ascospores; J. Paraphyses; K. Ascus apical apparatus (stained in Melzer’s reagent); L. Ascospore with germ slits; M. Ascospore in Indian ink; N. Culture on PDA. Scale bars: 0.2 mm (B–D); 100 µm (E); 10 µm (F–M).

##### Culture characteristics.

Ascospores germinated on PDA within 24 hours. Colonies reaching 55 mm diam., after 4 weeks at 24–27 °C on PDA. Colony surface white towards edges, dirty brown at centre, with densely mycelium, cottony, circular, velvety, smooth-edged, centre raised; reverse pale brown.

##### Material examined.

China • Yunnan Province, Ailaoshan National Nature Reserve (24°5'7.01"N, 101°31'30.44"E), altitude: 1169 m, on a dead palm branch, 15 September 2024, Changtao Lu, 2024ALS45 (GMB4902; GMBC4902); China • Yunnan Province, Ailaoshan National Nature Reserve (24°5'28.15"N, 101°31'45.23"E), altitude: 1172 m, on a dry palm branch, 16 September 2024, Changtao Lu, 2024ALS267 (GMB4951, GMBC4951).

##### Notes.

Our collections (GMB4902 and GMB4951) clustered with *M. citri* with strong support (100BS/1.00PP) (Fig. 1). *Melanographium citri* has previously been known only from the asexual state (Ellis 1963; Zhang et al. 2024), whereas our collections represent solely the sexual state, which precludes direct morphological comparison. Molecular comparison between our collections (GMB4902, GMB4951) and the reference specimen of *M. citri* (HKAS 115665, Zhang et al. (2024)) revealed minimal differences, 1.1% (6/536 bp) in ITS, 0.4% (4/853 bp) in LSU, 0.3% (3/1009 bp) in *rpb2* and 0.2% (2/934 bp) in *tef-1α*. Given the high sequence similarity (> 99% in both *rpb2* and *tef1-α*), along with prior documentation of this species from south-western China (Zhang et al. 2024), we identify our collections as *Melanographium citri*. Ecologically, *M. citri* has so far been reported exclusively from palm substrates (Arecaceae), including decaying petioles of *Trachycarpus fortunei* and the wood or bark of *Cocos*, *Elaeis*, *Phoenix*, *Sabal* and *Trachycarpus* spp. (Ellis 1963; Zhang et al. 2024), whereas our specimens were isolated from decaying bamboo culms (Poaceae), representing a novel substrate record. The ecological difference in host substrate suggests expansion of the known ecological range of this species.

With this report, we present the first documentation of the sexual state in *Melanographium*, characterised by globose to subglobose ascomata, eight-spored, clavate to cylindrical, non-amyloid asci and fusiform, dark brown ascospores with rounded ends, a short germ slit and a mucilaginous sheath. Phylogenetically, *Melanographium* species forms a distinct clade at the basal of Pallidoperidiaceae. It differs from other genera in Pallidoperidiaceae (Sugita et al. 2024; Habib et al. 2025) by its fusiform ascospores with a short germ slit at the centre and relatively wider asci with non-amyloid apical apparatus. *Amphigermslita* species possess amyloid, wedge-shaped apical apparatuses and ellipsoid to fusoid ascospores with short germ slits at both ends (Sugita et al. 2024; Habib et al. 2025). *Crassipseudostroma* is distinguished by fusiform to ellipsoid ascospores with mucilaginous pads and a full-length germ slit. *Minuticlypeus* and *Nigropunctata* have cylindrical asci with amyloid apical apparatuses and ellipsoid to oblong ascospores with germ slits extending the full length of the spore (Sugita et al. 2024).

#### ﻿ Nigropunctata puerzhenensis

Taxon classificationFungiXylarialesPallidoperidiaceae

C.T. Lu, K. Habib & Q. R. Li sp. nov.

8587E835-1B66-576C-B76E-A2768E39C003

MB859335

Fig. 3

##### Etymology.

The specific epithet “*puerzhenensis*” refers to Puer Village, where the holotype specimen was collected.

##### Type.

China • Yunnan Province, Zhaotong City, Puer Town (28°17'35.32"N, 103°59'28.57"E), altitude: 1154 m, on dead culms of bamboo, 21 August 2024, Changtao Lu, 2024PEZ5 (GMB4904, holotype, no culture was obtained); *ibid*KUN-HKAS 146984, isotype.

##### Description.

Saprobic on dead culms of bamboo. ***Sexual morph***: Ascomata 870–1230 µm wide, 760–910 µm high, immersed, solitary, scattered, appearing as small black dots, with minute clypeus, in cross-section globose to subglobose with a flattened base. Ostioles centric, slightly papillate, black, flush with the surface of the host. Peridium 15–36.5 µm thick, comprised of two layers; outer layer composed of thick-walled, dense, dark brown, cells of ***textura angularis***; inner layer hyaline cells of ***textura angularis***. Paraphyses 2–6 µm (x̄ = 4 µm, n = 30) wide, longer than the asci, numerous, filamentous, guttulate. Asci 159–233 × 10–26 µm (x̄ = 190 × 17.5 µm, n = 30) 8-spored, unitunicate, cylindrical, short-pedicellate, apically rounded, with a J+, discoid apical apparatus, 0.5–1.7 µm high, 2.5–4.5 µm wide (x̄ = 1.0 × 3.5 µm, n = 30). Ascospores 13–20 × 5.6–7.5 µm (x̄ = 16 × 6.5 µm, n = 30), uniseriate, unicellular, brown to dark brown, oblong to broadly ellipsoidal, with a germ slit, straight, along the entire spore length, surrounded by thick 2–6 µm (x̄ = 3 µm, n = 30) mucilaginous sheath. ***Asexual morph***: Undetermined.

**Figure 3.**
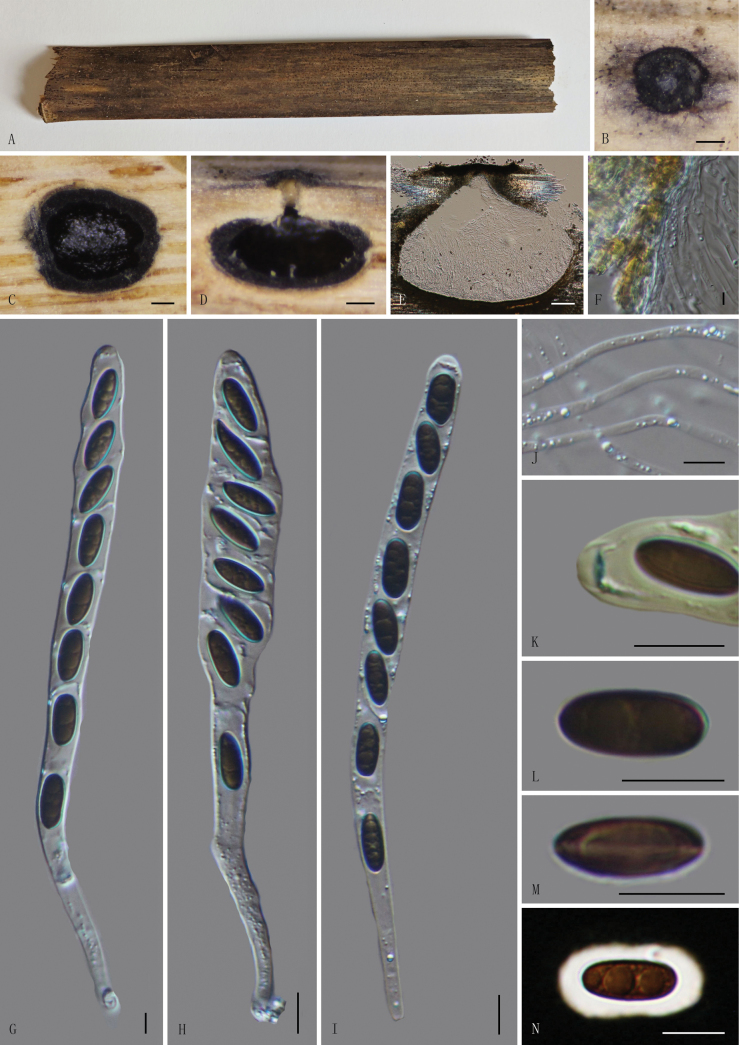
*Nigropunctata puerzhenensis* (GMB4904, holotype). A. Material; B. Ostiole appearance at the host surface; C. Cross-section of ascoma; D, E. Longitudinal sections of ascomata; F. Peridium; G–I. Asci in distilled water; J. Paraphyses; K. J+ apical apparatus bluing in Melzer’s reagent; L–N. Ascospores; (M. Ascospore with germ slit. N. Ascospore in Indian ink). Scale bars: 0.2 mm (B–D); 100 µm (E); 10 µm (F–N).

##### Paratype.

China • Yunnan Province, Zhaotong City, Puer Town (28°17'56.77"N, 103°59'47.34"E), altitude: 1168 m, on dead culms of bamboo, 21 August 2024, Changtao Lu, 2024PEZ11 (GMB4953, paratype).

##### Notes.

Morphologically and phylogenetically, *Nigropunctata puerzhenensis* is closely related to *N. nigrocircularis*. Both species share an amyloid ascus apical apparatus and ascospores surrounded by a thick mucilaginous sheath. However, *N. nigrocircularis* differs from *N. puerzhenensis* by having smaller ascomata (450–535 × 455–560 μm vs. 870–1230 × 760–910 μm), a thinner peridium (11–20 μm vs. 15–36.5 μm thick) and shorter asci (125–170 μm vs. 159–233 μm in length) (Samarakoon et al. 2022). Comparative analysis of nucleotide base pairs showed that *N. puerzhenensis* differs from *N. nigrocircularis* (MFLU 19-2130) by 3.2% (11/342) in the ITS locus, 1.4% (13/928) in the LSU locus, 1.4% (12/889) in the *tef-1a* gene and 1.0% (4/422) in the *tub2* gene. *Nigropunctata puerzhenensis* can be distinguished from all currently known species in the genus (Habib et al. 2025) by its larger asci. Comparison with other species of the genus is presented in Table 2.

**Table 2.** Comparison of the main morphological characteristics of the species of *Nigropunctata*.

Species	Ascomata (µm)	Peridium (µm)	Paraphyses (µm)	Asci (µm)	Apical apparatus (µm)	Ascospores (µm)	Mucilaginous sheath (µm)	Germ slit	Reference
*Nigropunctata bambusicola*	285–315 × 260–340	10–18	3–5	95–140 × 9.5–12.5	4–4.8 × 1.7–2	13.5–17 × 5.5–9.5	2–6	along the entire spore length	Samarakoon et al. (2022)
*Nigropunctata chiangraiensis*	400–480 × 300–380	200–240	2–3.5	80–160 × 9–15	4.5–7 wide	13.5–19 × 5–7	3–5	along the entire spore length	Tun et al. (2024)
*Nigropunctata chinensis*	580–670 × 355–420	55–80	1.8–3	144–180 × 12–16	non-amyloid	15–17.5 × 9–11.5	lack	lack germ slit	Habib et al. (2025)
*Nigropunctata complanata*	390–450 × 340–400	5–17.5	2.5–4.5	130–175 × 13–20	4.5–5 × 2.5–3	14.5–19.5 × 7.5–10	N/A	along the entire spore length	Sugita et al. (2024)
*Nigropunctata conspicosa*	N/A	N/A	2–5	126–145 × 10–11	N/A	14–19 × 7–8	5–12	along the entire spore length	Cao et al. (2025)
*Nigropunctata hydei*	485–575 × 400–520	16.5–31	3.5–5.6	150–185 × 11.5–16.5	3.8–5.2 × 1.8–3.4	13.5–18 × 7–10	N/A	lack germ slit	Samarakoon et al. (2023)
*Nigropunctata khalidii*	608–782 × 762–830	11–16	3.6–5.4	146–173 × 8.6–13.6	2.7–3.7 × 2.3–3.1	14.8–18 × 6.3–9	3.4–4.4	lack germ slit	Li et al. (2024)
*Nigropunctata liuzhouensis*	510–600 × 450–550	15–35	3–4	109–157.5 × 9.2–15.5	3.2–4.5 × 1.5–3	12.4–22 × 6.5–9.7	3–6.4	lack germ slit	Habib et al. (2025)
*Nigropunctata nigrocircularis*	450–535 × 455–560	11–20	2.5–4	125–170 × 8–10.5	3.2–3.6 × 1.2–2.5	12.5–19 × 5–8	3–4.5	along the entire spore length	Samarakoon et al. (2022)
***Nigropunctata puerzhenensis***	870–1230 × 760–910	15–36.5	2–6.2	159–233 × 10–26	2.5–4.5 × 0.5–1.7	13–20 × 5.6–7.5	2–6	along the entire spore length	**The study**
*Nigropunctata saccata*	365–440 × 290–370	10–15	2–3.3	90–130 7–10	No data	10.5–18 × 4.5–8	lack	along the entire spore length	Samarakoon et al. (2023)
***Nigropunctata shiwandashanensis***	610–1180 × 490–730	8–12.5	2.5–7.3	167–220 × 10–24	3–5 × 1–2	15–24 × 5.5–9	2–5	along the entire spore length	**The study**
*Nigropunctata thailandica*	615–830 × 770–965	10–14	2.8–5.5	120–155 × 11–16	4.5–6 wide	15–18.5 × 7–11.5	2–4.5	along the entire spore length	Samarakoon et al. (2022)

Notes: “N/A”: indicates not available.

#### ﻿ Nigropunctata shiwandashanensis

Taxon classificationFungiXylarialesPallidoperidiaceae

C.T. Lu, K. Habib & Q. R. Li sp. nov.

385FBE71-6B41-5C87-8FBA-70B5083D3E91

MB859334

Fig. 4

##### Etymology.

The specific epithet “*shiwandashanensis*” refers to Shiwandashan National Nature Reserve, where the holotype specimen was collected.

##### Type.

China • Guangxi Zhuang Autonomous Region, Shiwandashan National Nature Reserve (21°58'17.88"N, 108°2'52.22"E), altitude: 315 m, on dead culms of bamboo, 22 September 2024, Changtao Lu, 2024SWS82 (GMB4903, holotype; GMBC4903, ex-type); *ibid*KUN-HKAS 146985, isotype.

##### Description.

Saprobic on dead culms of bamboo. ***Sexual morph***: Ascomata 610–1180 µm wide, 490–730 µm high, immersed, solitary, scattered, appearing as small black dots, in cross-section globose to subglobose with a flattened base. Ostioles centric, slightly papillate, black, covered with black, thick-walled clypeus. Peridium 8–12.5 µm thick, comprised of two layers; outer layer composed of thick-walled, dense, dark brown cells of ***textura angularis***; inner layer hyaline cells of ***textura angularis***. Paraphyses 2.5–7.3 µm (x̄ = 6.1 µm, n = 30) wide, longer than the asci, numerous, filamentous, contain white intracellular material. Asci 167–220 × 10–24 µm (x̄ = 195.5 × 17.5 µm, n = 30), 8-spored, unitunicate, cylindrical, short-pedicellate, apically rounded, with a J+, discoid apical apparatus, 1–2 µm high, 3–5 µm wide (x̄ = 1.5 × 4 µm, n = 30). Ascospores 15–24 × 5.5–9 µm (x̄ = 19.7 × 7.0 µm, n = 30), uniseriate, unicellular, brown to dark brown, oblong to broadly ellipsoidal, surrounded by 2–5 µm (x̄ = 3.7 µm, n = 30) thick mucilaginous sheath, with a straight germ slit along the entire spore length. ***Asexual morph***: Undetermined.

**Figure 4.**
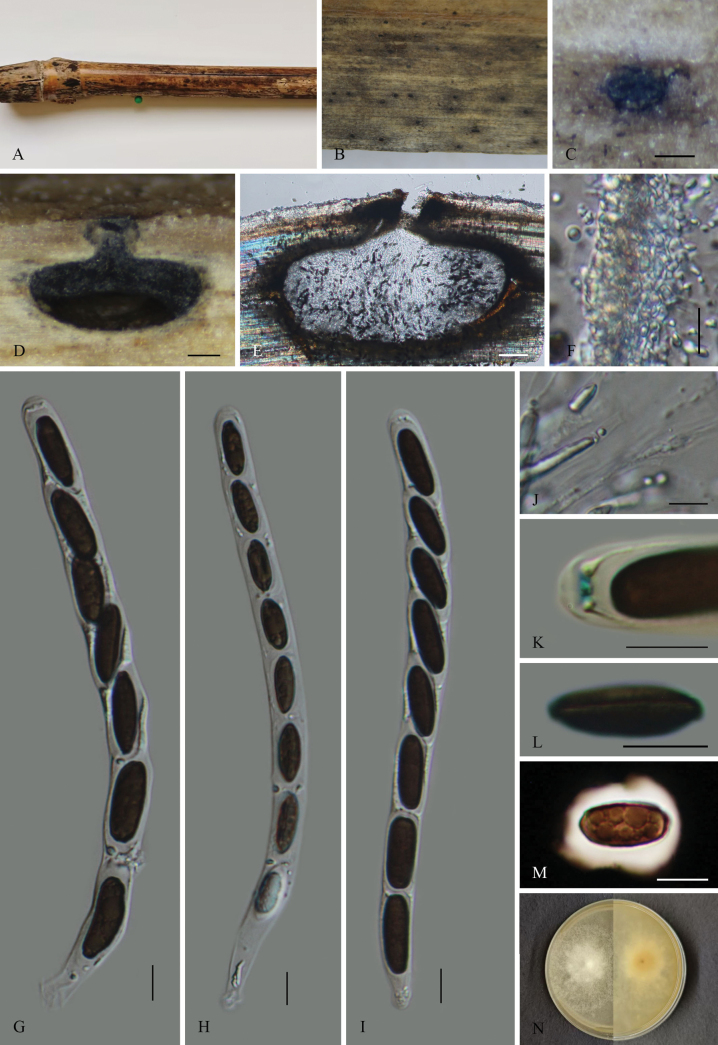
*Nigropunctata shiwandashanensis* (GMB4903, holotype). A. Bamboo host; B, C. Ostioles appearance at the host surface; D, E. Longitudinal sections of ascomata; F. Peridium; G–I. Asci in distilled water; J. Paraphyses; K. J+ apical apparatus bluing in Melzer’s reagent; L–M. Ascospores (L. Ascospores with germ slit. M. In Indian ink); N. Culture on PDA. Scale bars: 0.2 mm (B–D); 100 µm (E); 10 µm (F–M).

##### Culture characteristics.

Ascospores germinated on PDA within 36 hours. Colonies grow fast, reaching 2 cm in 1 week at 24–27 °C. Colony surface white, velvety to hairy, thin, nearly circular; reverse, pale yellowish.

##### Paratype.

China • Guangxi Zhuang Autonomous Region, Shiwandashan National Nature Reserve (21°59'37.26"N, 108°2'44.36"E), altitude: 326 m, on dead culms of bamboo, 22 September 2024, Changtao Lu, 2024SWS109 (GMB4952, GMBC4952).

##### Notes.

In the phylogram (Fig. 1), *Nigropunctata shiwandashanensis* and *N. hydei* form a sister clade with strong support value (91BS/1PP). Morphologically, *N. shiwandashanensis* can be distinguished from *N. hydei* by its larger ascomata (613–1180 × 490–730 µm vs. 485–575 × 400–520 µm), thinner peridium (8.3–12.5 µm vs. 16.5–31 µm), larger asci (167–220 × 10–24 µm vs. 150–185 × 11.5–16.5 µm) and longer ascospores (15–24 × 5.5–9 µm vs. 13.5–18 × 7–10 µm). In addition, the ascospores of *N. hydei* lack a germ slit (Samarakoon et al. 2023), whereas *N. shiwandashanensis* possesses a full-length, straight germ slit. Comparative analysis of nucleotide base pairs showed that *Nigropunctata shiwandashanensis* differs from *N. hydei* by 10.5% (51/487) in the ITS locus, 2.6% (25/946) in the LSU locus and 7.5% (71/951) in the *rpb2* gene. Comparison with other morphologically similar species is presented in Table 2.

## ﻿Discussion

All known *Nigropunctata* species are bambusicolous saprobes exclusively known from their sexual morph. To date, no asexual state has been observed either in ascospore-derived cultures or in natural substrates. The genus has a distribution in East and Southeast Asia, with the majority of species reported from south-western China (Li et al. 2024; Habib et al. 2025; Liu et al. 2025). Prior to this study, 11 species were known in the genus, our work increasing this number to 13.

Morphologically, species within the genus *Nigropunctata* are separated, based on the size of ascomata, asci and ascospores, as well as the presence or absence of a germ slit or mucilaginous sheath. However, these differences are often subtle and can be challenging to distinguish, especially for characters like ascomata size which may vary with maturity. Ascospore size differences between species are also often minor, differing by only 1 or 2 µm. Accurate identification often requires a combined approach utilising both morphology and DNA sequence analysis, as highlighted by Habib et al. (2025).

*Melanographium* has been classified as *incertae sedis* within Xylariales. In both our phylogenetic analysis and previous studies (Samarakoon et al. 2022; Sugita et al. 2024; Zhang et al. 2024; Zhou et al. 2024; Habib et al. 2025; Liu et al. 2025), *Melanographium* consistently forms a distinct monophyletic clade that is sister to Pallidoperidiaceae. Although this clade lacks strong statistical support, it consistently appears stable in our repeated phylogenetic analyses and in multiple independent studies (Samarakoon et al. 2022; Sugita et al. 2024; Zhang et al. 2024; Zhou et al. 2024; Habib et al. 2025; Liu et al. 2025). The low support values may indicate that current taxon sampling remains insufficient, emphasising the need for further research with expanded datasets. Sugita et al. (2024) did not include *Melanographium* in Pallidoperidiaceae due to the lack of a known sexual morph and the morphological differences of its asexual state (synnematous conidiomata and reniform, pigmented conidia) compared to the known sexual morphs of other Pallidoperidiaceae species. However, the sexual morph characteristics now identified for *Melanographium* (ascomata immersed, asci 8-spored, cylindrical, apical apparatus inamyloid, ascospores brown, fusiform, unicellular, surrounded by mucilaginous appendages, with a germ slit) aligns definitively with the characteristics of Pallidoperidiaceae. Therefore, based on its persistent phylogenetic placement as the sister lineage to Pallidoperidiaceae and the agreement of its sexual morphic characteristics with the family, we include *Melanographium* within Pallidoperidiaceae.

## Supplementary Material

XML Treatment for Melanographium

XML Treatment for Melanographium citri

XML Treatment for Nigropunctata puerzhenensis

XML Treatment for Nigropunctata shiwandashanensis

## ﻿Additional information

### Use of AI

No use of AI was reported.

### Author ORCIDs

Chang-Tao Lu https://orcid.org/0009-0009-8157-7365

Yu-Lin Ren https://orcid.org/0009-0003-9063-425X

Kamran Habib https://orcid.org/0000-0003-2572-0306

Qin-Fang Zhang https://orcid.org/0009-0003-9408-4988

Li-Li Liu https://orcid.org/0009-0003-8481-6137

Ji-Chuan Kang https://orcid.org/0000-0002-6294-5793

Xiang-Chun Shen https://orcid.org/0000-0002-4333-9106

Nalin N. Wijayawardene https://orcid.org/0000-0003-0522-5498

Salim S. Al-Rejaie https://orcid.org/0000-0002-9254-1087

Chanhom Loinheuang https://orcid.org/0009-0008-4852-9700

Qi-Rui Li https://orcid.org/0000-0001-8735-2890

Qing-De Long https://orcid.org/0000-0002-7720-2650

Abdallah M. Elgorban https://orcid.org/0000-0003-3664-7853

### Data availability

All of the data that support the findings of this study are available in the main text.
